# MiR-3613-3p affects cell proliferation and cell cycle in hepatocellular carcinoma

**DOI:** 10.18632/oncotarget.21745

**Published:** 2017-10-10

**Authors:** Donghui Zhang, Enqin Liu, Jian Kang, Xin Yang, Hong Liu

**Affiliations:** ^1^ Department of Infectious Disease, Linyi People's Hospital, Linyi 276000, China; ^2^ Department of Colorectal Surgery, Tai’an City Central Hospital, Tai’an 271000, China; ^3^ Culverhouse College of Commerce and Business Administration, The University of Alabama, Tuscaloosa, AL 35401, USA

**Keywords:** cell cycle, cell proliferation, hepatocellular carcinoma, hsa-miR-3613-3p

## Abstract

Hepatocellular carcinoma (HCC) is one of the most common types of malignant tumors with poor sensitivity to chemotherapy drugs and poor prognosis among patients. In the present study, we downloaded the original data from the Gene Expression Omnibus and compared gene expression profiles of liver cancer cells in patients with HCC with those of colon epithelial cells of healthy controls to identify differentially expressed genes (DEGs). After filtering target microRNAs (miRNA) from core DEGs, we cultured HepG2 cells *in vitro*, knocked down the miRNA and core mRNAs, and analyzed the effects. We found 228 differentially expressed genes between liver cancer tissue and healthy control tissue. We also integrated the protein-proteininteraction network and module analysis to screen 13 core genes, consisting of 12 up-regulated genes and 1 down-regulated gene. Five core genes were regulated hsa-miR-3613-3p, therefor we hypothesized that hsa-miR-3613-3p was a critical miRNA. After the transfection procedure, we found that changes in hsa-miR-3613-3p were the most obvious. Therefore, we speculated that hsa-miR-3613-3p was a main target miRNA. In addition, we transfected with si (BIRC5, CDK1, NUF2, ZWINT and SPC24), to target genes that can be targeted by miR-3613-3p. Our data shows that BIRC5, NUF2, and SPC24 may be promising liver cancer biomarkers that may not only predict disease occurrence but also potential personalized treatment options.

## INTRODUCTION

Hepatocellular carcinoma (HCC) is a standout amongst the most well-known threatening tumors. Patients with HCC have poor sensitivity to chemotherapy drugs, and the prognosis among patients is often poor [1, 2]. Selective intervention of cell signal transduction is becoming an effective treatment for malignant tumors. However, the molecular markers of HCC were controversial [3–5]. For the analysis of gene expression, high-throughput platforms such as microarray analysis, are becoming promising tools in the field of medical oncology with great clinical applications. Such clinical applications include molecular diagnosis, molecular classification of cancers, patient stratification, prognosis prediction, novel drug targets discovery, and tumor response prediction. The cell cycle is regulated by several factors with two main control points: one at the G1/S checkpoint, which controls the cells to enter the S phase (G1 phase detection point), and the other at the G2/M checkpoint, which controls the cells to enter the M phase (G2 detection point). Cell cycle dysregulation is a major hallmark of tumor cells. The ability of normal cells to undergo cell-cycle arrest after DNA damage is crucial for the maintenance of cell integrity. MicroRNAs (miRNAs) are known to play critical roles in pathogenesis of HCC. For example, miR-122 is a liver-specific microRNA that can inhibit HCC cell growth by inducing G2/M arrest. In HCC, miR-122 is frequently downregulated [6].

The events involved in cell reproduction are governed by oscillations in the activities of cyclin-dependent kinases (CDKs) [7]. Elevated expression of CDK inhibitors results in cell cycle arrest [8]. The inducible depletion of CDK1 activity from cancer cell lines causes accumulation of cells in the G2/M phase, and is accompanied by cell death in a cell line-dependent manner [9]. CDK1 depletion induces irreparable DNA damage, G2/M arrest, and cell death [10]. Baculoviral IAP Repeat Containing 5 (BIRC5) is preferentially expressed in human cancer cells and mediates cancer cell survival and tumor maintenance [11]. In human tumor tissue, BIRC5 has several characteristics, including a high expression level, a close association with proliferative activities, high transfer capacity, and resistance to chemotherapy. This indicates that BIRC5 may be a promising target for cancer therapy [12–14]. Introducing BIRC5 antisense oligonucleotides or BIRC5 inhibitors into cancer cells reduced BIRC5 expression in cells, inhibited cell proliferation, and lowered the cell apoptosis rate [15]. BIRC5 acts as an apoptosis inhibitor, plays, in part, an important role in carcinogenesis, and thus may serve as a promising target for antitumor therapy [16].

Cell proliferation can be regulated by many factors. In addition to the length of the cell cycle, cell DNA replication synthesis and cell proliferation can also be regulated by genes named cell proliferation-related antigens, such as PCNA and Ki67. PCNA is a nuclear protein that is widely expressed in the S phase of the cell cycle. Together with DNA polymerase cofactors, PCNA participates in the regulation of DNA synthesis [17]. Given that the S phase of the cell cycle is the stage where cells actively undergo DNA replication, the level of PCNA expression may reflect the cell proliferation activity. Ki67 is a marker that reflects tumor cell proliferation and can be used an indicator of tumor proliferation and the degree of malignancy.

Furthermore, NUF2 has been reported to play a role in tumorigenesis of various types of human cancers. Studies have shown that depletion of NUF2 by specific siRNAs inhibited cell proliferation. Silencing of NUF2 significantly inhibited the proliferation of cancer cells *in vitro*, through inducing cell cycle arrest at the G0/G1 phase [18]. SPC24 plays an essential role in coupling kinetochore to spindle microtubules and in the precise segregation of chromosomes during mitosis [19]. SiRNA-mediated silencing of SPC24 dramatically suppressed cell growth and increased apoptosis in HCC cells [20]. Specific siRNAs targeting ZW10 interactor (ZWINT), a known component of the kinetochore complex required for the mitotic spindle checkpoint, diminished effects of ZWINT on cell proliferation [21].

Thus, regulation of proteins that mediate critical events of the cell-cycle may represent a potent anti-tumor approach [22]. Studying the relation between cell cycle regulatory and tumorigenesis is a hot topic in the field of tumor research. In the present study, we obtained the original data from Gene Expression Omnibus (GEO) (http://www.ncbi.nlm.nih.gov/geo). Gene expression profiles from the cancer cells of patients with HCC were compared with epithelial cells of healthy colon to identify DEGs. We filtered target miRNAs of core DEGs, cultured HepG2 cells *in vitro*, knocked down miRNA and core mRNAs, and evaluated the effects caused by the target miRNA and core mRNAs. This this study will further gain further insights into the development of HCC at the molecular level and explores the potential of candidate biomarkers for diagnosis, prognosis, and drug discovery.

## RESULTS

### Identification of DEGs

**Figure 1:**
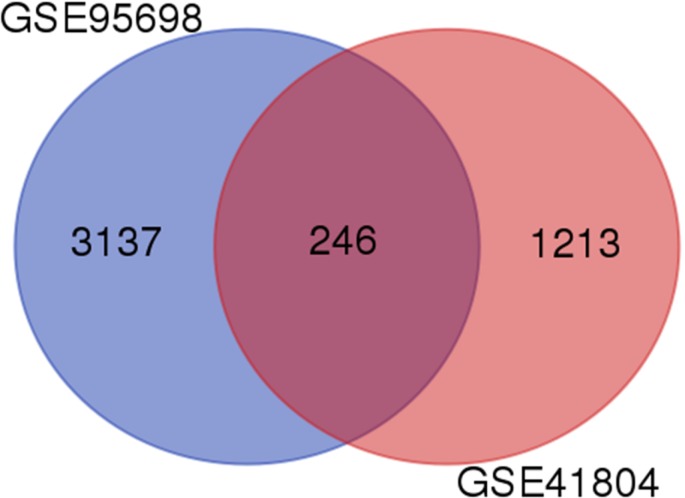
Identification of differentially expressed genes in mRNA expression profiling datasets Two human gene expression profiles (GSE95698 and GSE41804) contained data of cancer and non-cancerous tissues and were analyzed by Affymetrix Human Genome U133 Plus 2.0 Array.

A total of 3383 and 1459 DEGs were identified from GSE95698 and GSE41804 datasets, respectively. Among these two sets of DEGs, 246 genes presented similar trends in expression levels (Figure 1). When comparing liver cancer tissue to healthy control tissue, 71 genes were up-regulated genes while 157 genes were down-regulated.

### Functional and pathway enrichment analysis

All DEGs were uploaded into the online Database for Annotation, Visualization and Integrated Discovery (DAVID) software to identify overrepresented Gene Ontology (GO) categories and Kyoto Encyclopedia of Genes and Genomes (KEGG) pathways. The up-regulated genes were mainly involved in biological processes (BP) associated with cellular responses to amino acid stimuli, positive regulation of exit from mitosis, microtubule-based movement, regulation of cell cycle and translesion synthesis. The down-regulated DEGs were enriched in oxidation-reduction processes, cellular response to cadmium ions, complement activation, response to estradiol and complement activation, and lectin pathways. Regarding the cellular component (CC), the up-regulated DEGs were enriched in the cytoplasm, nuclei, caveolae, collagen trimers, and ndc80 complexes. The down-regulated DEGs were enriched in the extracellular region, blood microparticles, extracellular space, integral components of the plasma membrane and the organelle membrane. In addition, GO molecular function (MF) analysis displayed that the up-regulated DEGs were significantly enriched in chromatin binding, phosphatidylinositol phospholipase C activity, ATP binding, and cysteine-type endopeptidase inhibitor activity. Down-regulated DEGs were enriched in serine-type endopeptidase activity, carbohydrate binding, monooxygenase activity, iron ion binding, and heparin binding (Table 1).

**Table 1:** Gene ontology analysis of differentially expressed genes

	Category	No	Item name	Gene number	P value
Up-regulated	BP_DIRECT	GO:0071230	cellular response to amino acid stimulus	3	1.28E-02
		GO:0031536	positive regulation of exit from mitosis	2	2.13E-02
		GO:0007018	microtubule-based movement	3	2.91E-02
		GO:0051726	regulation of cell cycle	3	3.18E-02
		GO:0019985	translesion synthesis	2	3.81E-02
	CC_DIRECT	GO:0005737	cytoplasm	22	9.89E-04
		GO:0005634	nucleus	20	2.52E-03
		GO:0005901	caveola	3	1.20E-02
		GO:0005581	collagen trimer	3	1.32E-02
		GO:0031262	Ndc80 complex	2	1.66E-02
	MF_DIRECT	GO:0003682	chromatin binding	5	2.60E-02
		GO:0004435	phosphatidylinositol phospholipase C activity	2	6.02E-02
		GO:0005524	ATP binding	9	7.07E-02
		GO:0004869	cysteine-type endopeptidase inhibitor activity	2	7.47E-02
Down-regulated	BP_DIRECT	GO:0055114	oxidation-reduction process	17	2.90E-05
		GO:0071276	cellular response to cadmium ion	4	3.39E-04
		GO:0006956	complement activation	6	7.51E-04
		GO:0032355	response to estradiol	6	9.20E-04
		GO:0001867	complement activation, lectin pathway	3	1.37E-03
	CC_DIRECT	GO:0005576	extracellular region	38	1.24E-09
		GO:0072562	blood microparticle	10	3.00E-06
		GO:0005615	extracellular space	28	4.68E-06
		GO:0005887	integral component of plasma membrane	25	2.36E-04
		GO:0031090	organelle membrane	5	4.77E-03
	MF_DIRECT	GO:0004252	serine-type endopeptidase activity	10	2.37E-04
		GO:0030246	carbohydrate binding	8	1.10E-03
		GO:0004497	monooxygenase activity	5	1.25E-03
		GO:0005506	iron ion binding	7	1.56E-03
		GO:0008201	heparin binding	7	1.96E-03

If there were more than five terms enriched in this category, top five terms were selected according to P value. Count: the number of enriched genes in each term.

Moreover, four KEGG pathways were over-represented in the cell cycle, p53 signaling pathway, protein digestion and absorption, amoebiasis, and the thyroid hormone signaling pathway. Down-regulated DEGs were enriched in complement and coagulation cascades, metabolic pathways, chemical carcinogenesis, tryptophan metabolism and biosynthesis of antibiotics (Table 2).

**Table 2:** KEGG pathway analysis of differentially expressed genes

	ID	Description	Gene count	P value	Genes
Up-regulatde	04110	Cell cycle	4	6.10E-03	CCNB1, CDK1, CDKN2A, PTTG1
	04115	p53 signaling pathway	3	1.69E-02	CCNB1, CDK1, CDKN2A
	04974	Protein digestion and absorption	3	2.73E-02	COL4A1, COL15A1, COL1A2
	05146	Amoebiasis	3	4.19E-02	COL4A1, COL1A2, PLCB1
	04919	Thyroid hormone signaling pathway	3	4.70E-02	NOTCH3, PLCE1, PLCB1
Down-regulated	hsa04610	Complement and coagulation cascades	7	1.16E-04	F11, C8A, C9, FGA, MASP1, F2, PLG
	hsa01100	Metabolic pathways	26	1.53E-03	NDST3, UPP2, ADH1B, GCH1, HK3, ST3GAL6, HAAO, CDA, RGN, DMGDH, AADAT, ST6GAL2, ASMT, CYP2C8, NAT2, HAL, CYP1A2, CYP2E1, GRHPR, DBH, PCK2, TAT, ACSM3, CTH, HMGCS2, UROC1
	hsa05204	Chemical carcinogenesis	6	2.00E-03	CYP3A43, CYP2C8, NAT2, ADH1B, CYP2E1, CYP1A2
	hsa00380	Tryptophan metabolism	4	1.01E-02	AADAT, ASMT, HAAO, CYP1A2
	hsa01130	Biosynthesis of antibiotics	7	3.20E-02	AADAT, CTH, HMGCS2, HK3, RGN, PCK2, TAT

If there were more than five terms enriched in this category, top five terms were selected according to P value. Count: the number of enriched genes in each term.

### Protein-protein interaction network construction and modules selection

**Figure 2:**
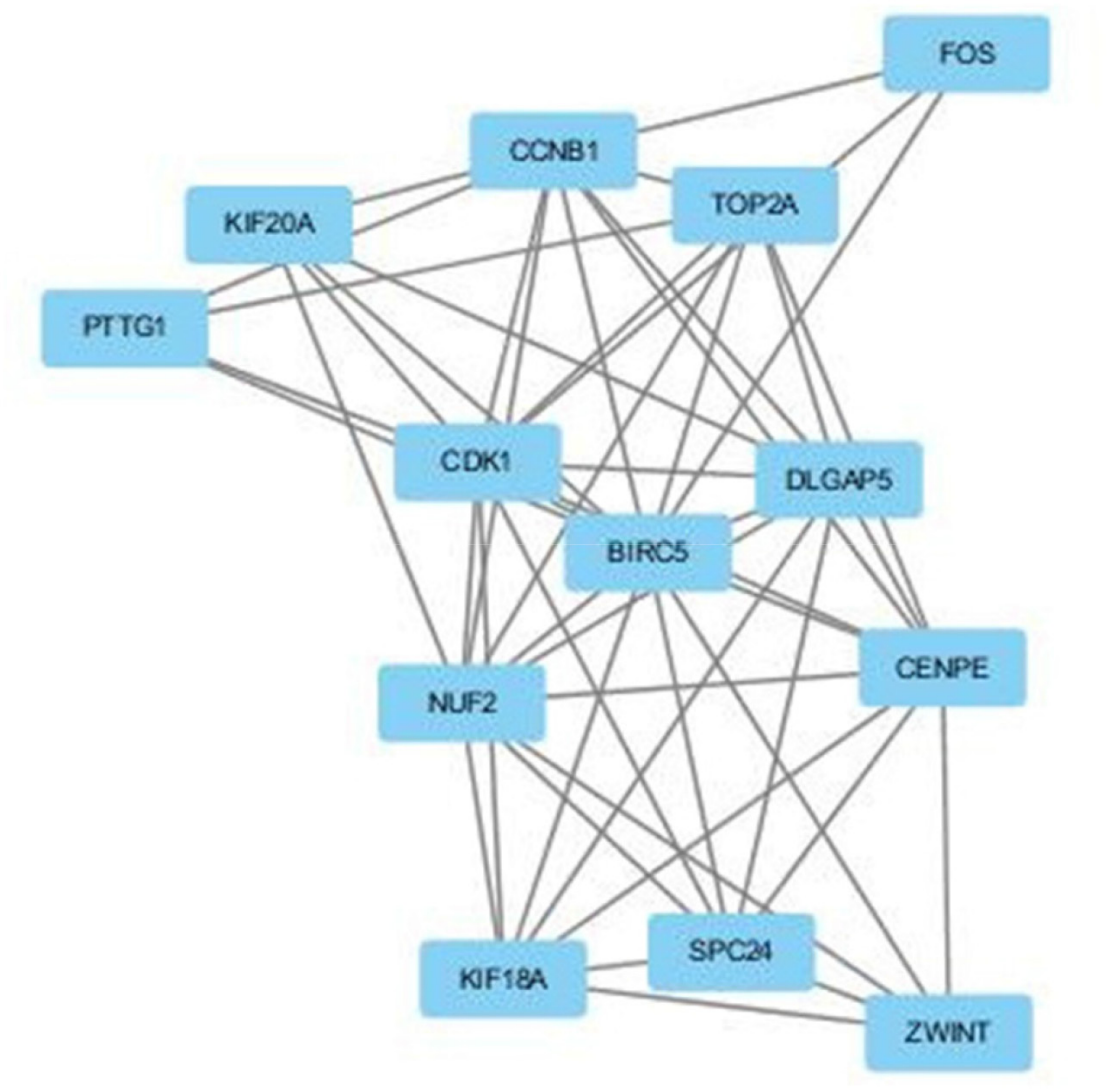
The protein–protein interaction (PPI) network of DEGs using Molecular Complex Detection (MCODE) The PPI network of DEGs was constructed using the Search Tool for the Retrieval of Interacting Genes (STRING, http://string-db.org) database and was visualized using Cytoscape (http://www.cytoscape.org/index.html). MCODE was performed to screen modules of the PPI network with a degree cutoff = 2, node score cutoff = 0.2, K-Core = 2, and Depth from Seed = 100.

A significant module was obtained from the protein-protein interaction (PPI) network of DEGs using Molecular Complex Detection (MCODE) (Figure 2), including 12 up-regulated genes and 1 down-regulated gene.

Functional and KEGG pathway enrichment analysis revealed that the genes in this module were mainly associated with the cell cycle, colorectal cancer, p53 signaling pathway, progesterone-mediated oocyte maturation, oocyte meiosis, hepatitis B, and herpes simplex infection (Table 3).

**Table 3:** KEGG pathway analysis of core genes

ID	Description	Gene count	P value	Genes
04110	Cell cycle	3	1.91E-03	CCNB1, CDK1, PTTG1
05210	Colorectal cancer	2	3.55E-02	FOS, BIRC5
04115	p53 signaling pathway	2	3.78E-02	CCNB1, CDK1
04914	Progesterone-mediated oocyte maturation	2	4.98E-02	CCNB1, CDK1
04114	Oocyte meiosis	2	6.17E-02	CDK1, PTTG1
05161	Hepatitis B	2	8.12E-02	FOS, BIRC5
05168	Herpes simplex infection	2	9.77E-02	CDK1, FOS

### DEG-mRNA pairs

A total of 13 differentially expressed mRNAs were screened, consisting of 12 up-regulated and 1 down-regulated mRNAs. Results indicated that hsa-miR-3613-3p was common among 5 up-regulated genes, including NUF2, BIRC5, CDK1, ZWINT, and SPC24.

### Core genes overexpressed in human liver cancer tissues

**Figure 3:**
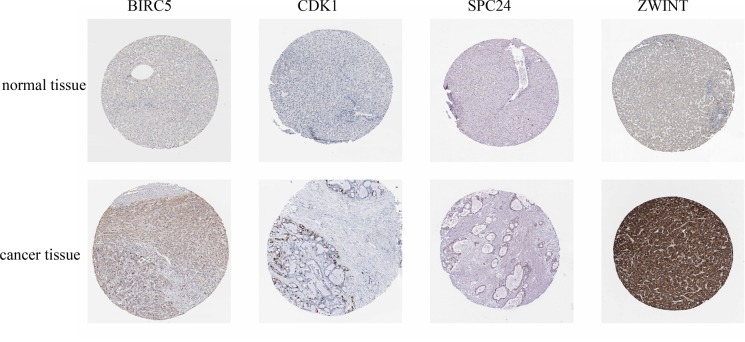
Immunohistochemistry of core genes in normal and cancerous tissue Protein expression of core genes in liver cancer tissue and normal tissue was determined from the human protein atlas (www.proteinatlas.org).

To identify the core genes that are targeted by hsa-miR-3613-3p, we first analyzed hsa-miR-3613-3p protein expression in clinical specimens from the human protein atlas. We found that BIRC5, CDK1, NUF2, ZWINT, and SPC24 were highly expressed in liver cancer tissues, whereas expression was weak in normal tissues (Figure 3).

### Expression of mRNA of these common differentially expressed genes in tissues

**Figure 4:**
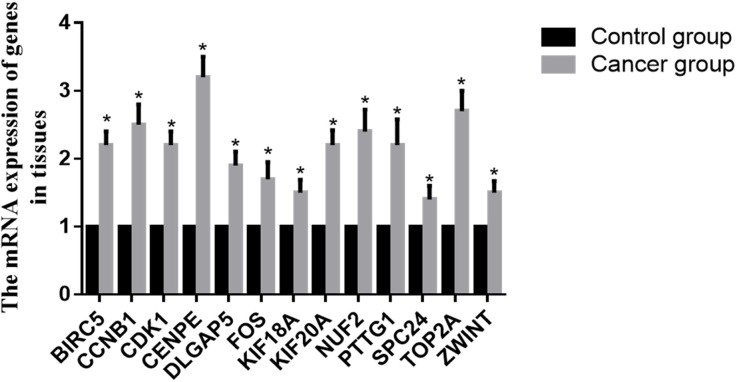
mRNA expression of core genes in tissue Data are presented as the mean ± SD. Compared with controls: ^*^P < 0.05.

The mRNA expression levels of these core genes were consistent with the results of our bioinformatics analysis (Figure 4). Compared with the control group, the mRNA expression of BIRC5, CCNB1, CDK1, CENPE, DLGAP5, KIF18A, KIF20A, NUF2, FOS, PTTG1, SPC24, TOP2A, and ZWINT displayed different levels of upregulation (P<0.05).

### Hsa-miR-3613-3p directly targeted the 3′UTR of BIRC5, CDK1, NUF2, ZWINT, and SPC24 in HepG2 cells

**Figure 5:**
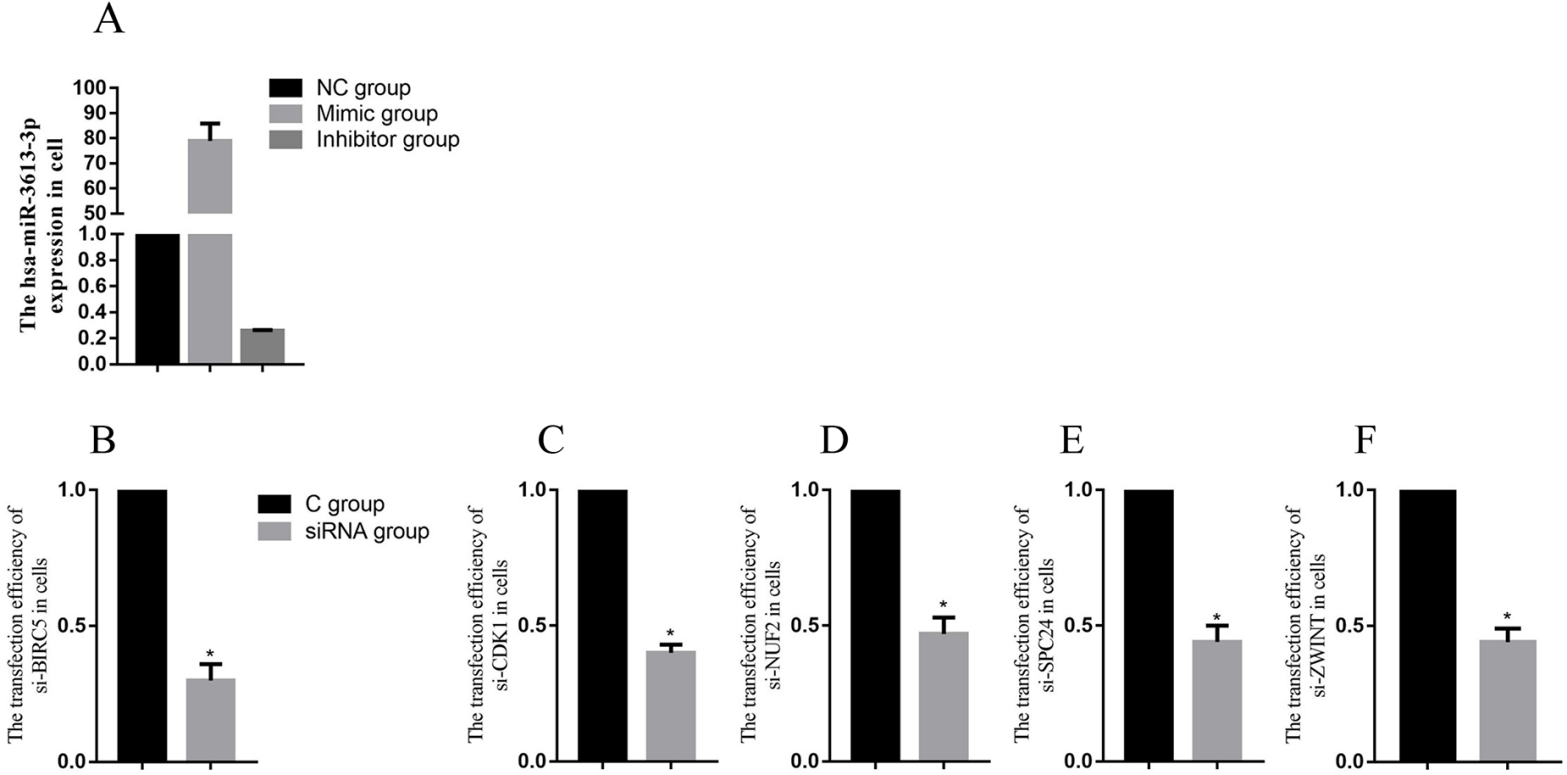
The transfection efficiency of miRNA and core genes Data are presented as the mean ± SD. Compared with controls: ^*^P < 0.05.

**Figure 6:**
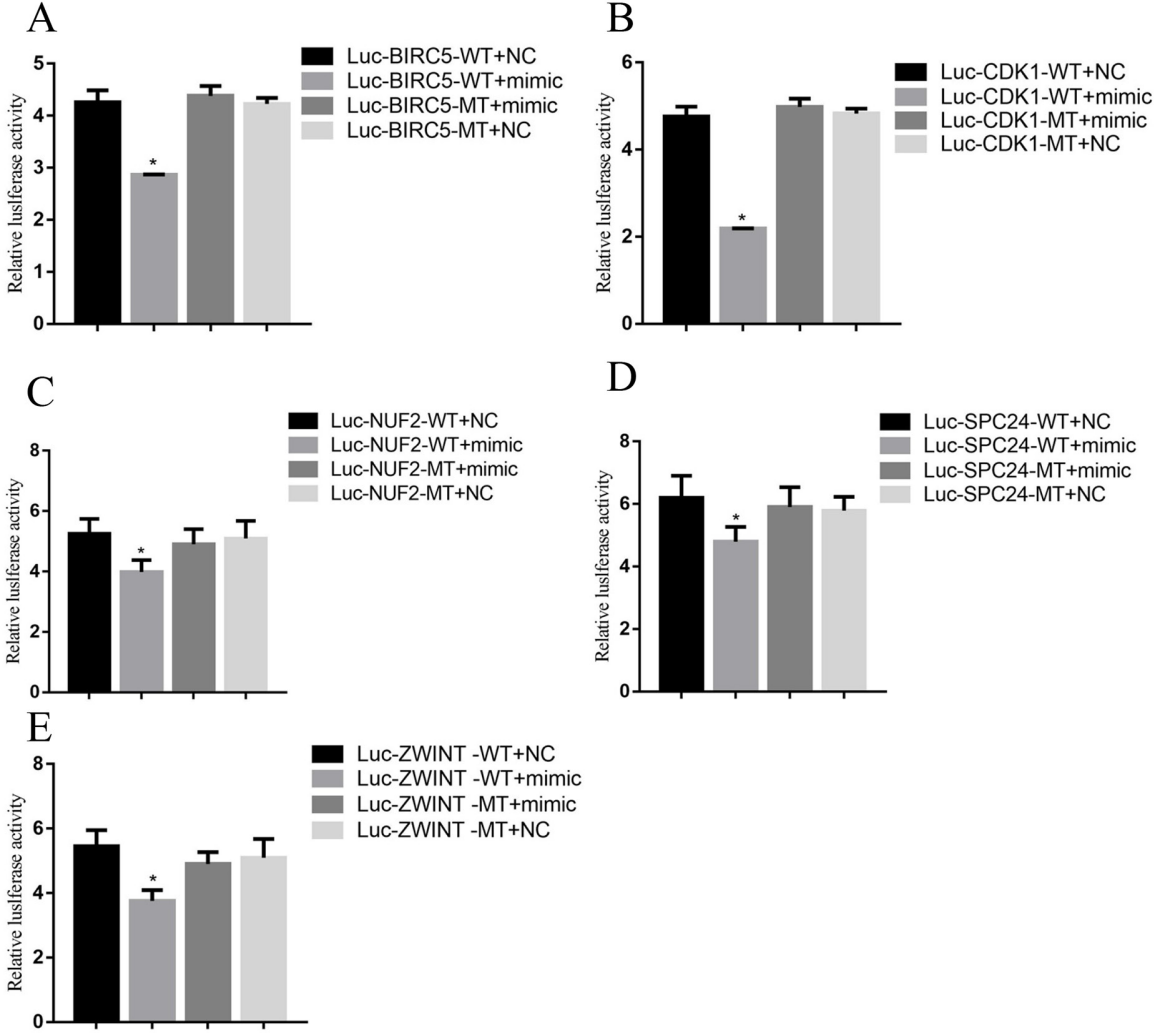
Luciferase reporter assay Data are presented as the mean ± SD. Compared with controls: ^*^P < 0.05.

To demonstrate a direct interaction between miR-3613-3p and core genes, such as BIRC5, CDK1, NUF2, ZWINT and SPC24, we first determined the transfection efficiency of mir-3613-3p, siBIRC5, siCDK1, siNUF2, siZWINT, and siSPC24 (Figure 5). Next, reporter gene vectors were transfected into HepG2 with miR-3613-3p mimics or negative controls. After 24 hours, luciferase activity in HepG2 cells that were co-transfected with a pMIR-BIRC5 vector and miR-3613-3p mimic decreased to 30.0% compared with control miRNA. Luciferase activity of cells co-transfected with pMIR-CDK1 vector and miR-3613-3p mimic decreased to 40.0% compared with control miRNA. Cells co-transfected with the pMIR-NUF2 vector and miR-3613-3p mimic showed a reduced luciferase activity of 47% compared to control miRNA. Cells co-transfected with the pMIR-ZWINT vector and miR-3613-3p mimic decreased to 44% compared with control miRNA. Cells co-transfected with pMIR-SPC24 vector and miR-3613-3p mimic decreased to 44% compared with control miRNA (Figure 6). When the miR-3613-3p binding site was mutated in the 3′UTR region of the core genes, luciferase activity was no longer inhibited by miR-3613-3p mimics. These findings demonstrated that hsa-miR-3613-3p reduced the expression of BIRC5, CDK1, NUF2, ZWINT, and SPC24 mRNA and affected cell proliferation.

### The effects of hsa-miR-3613-3p and core genes on cell proliferation

**Figure 7:**
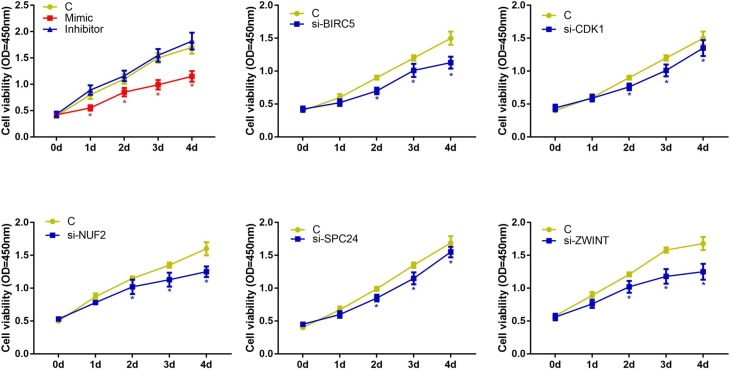
Cell viability detected by CCK8 Data are presented as the mean ± SD. Compared with controls: ^*^P < 0.05.

We examined the effects of hsa-miR-3613-3p and core genes on HepG2 cell proliferation by performing CCK-8 assays. The CCK-8 assay demonstrated that hsa-miR-3613-3p mimics inhibited proliferation from 1 day to 4 days (Figure 7). Furthermore, we used the siRNA approach to silence the core genes BIRC5, CDK1, NUF2, ZWINT, and SPC24 in HepG2 cells, and examined the proliferation ability of the cells. Our data indicated that when treated with siBIRC5, the cell viability at 2-4 days decreased compared with that of 0 days. Cells transfected with siCDK1, siNUF2, siZWINT, or siSPC24, showed a reduction in cell viability at 2-4 days after transfection compared with that at day 0. These findings demonstrated that hsa-miR-3613-3p and core genes affected cell proliferation.

**Figure 8:**
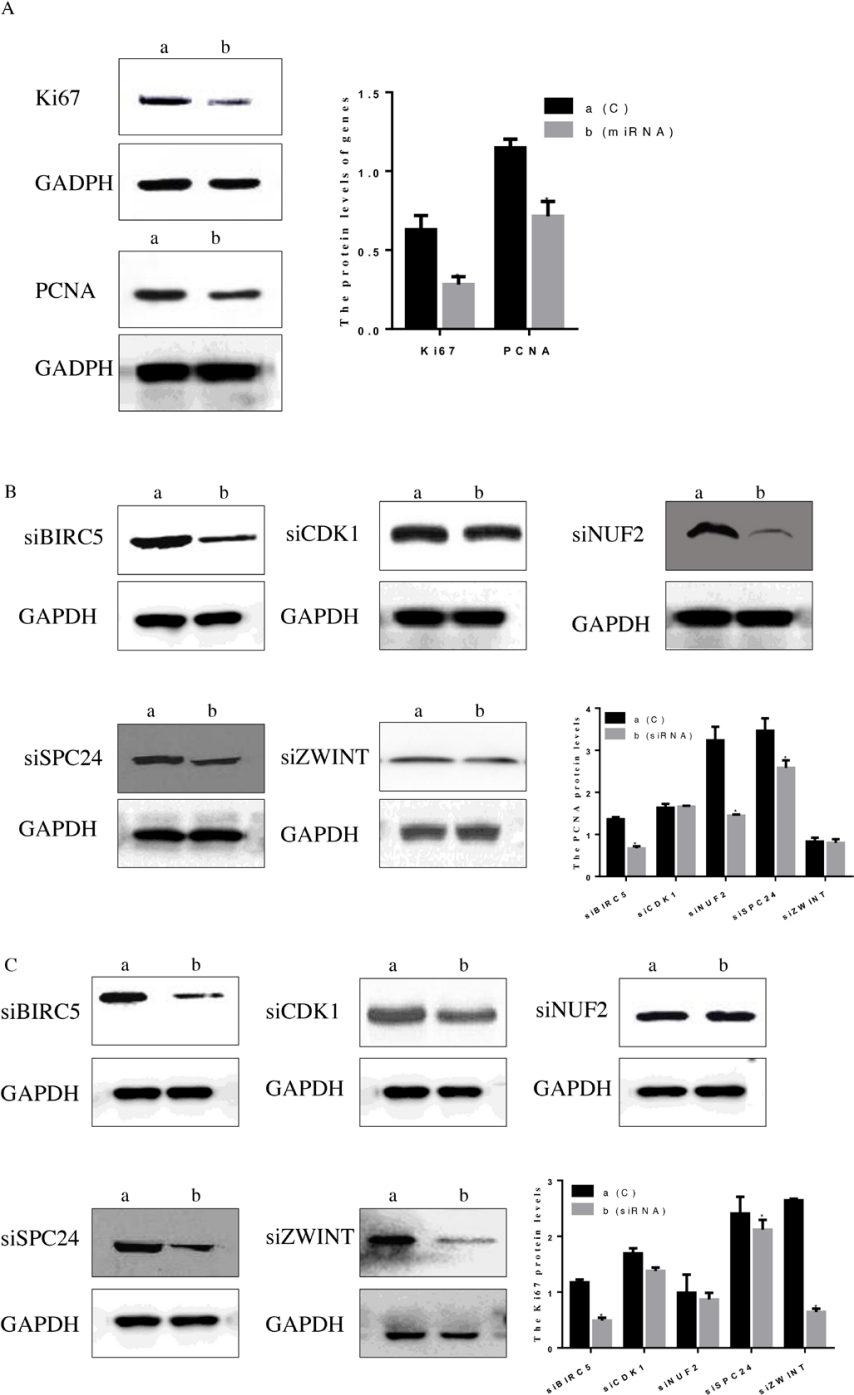
Protein level of Ki67 and PCNA after treatment with miRNA mimic and simRNA (core mRNA) **(A)** Treatment with miRNA, **(B)** PCNA protein level after treatment with simRNA, **(C)** Ki67 protein level after treatment with simRNA. Data are presented as the mean ± SD. Compared with controls: ^*^P < 0.05.

Except for CCK8, we evaluated the protein levels of Ki67 and PCNA in HepG2 cells after transfection with hsa-miR-3613-3p mimics and siRNA (Figure 8A). The data indicated that when cells were treated with hsa-miR-3613-3p mimics, the levels of Ki67 and PCNA both decreased compared with negative control group (P < 0.05). Moreover, The Ki67 and PCNA protein level also decreased when cells were transfected with siBIRC5, siCDK1 or siZWINT (P < 0.05) (Figure 8B, 8C).

**Figure 9:**
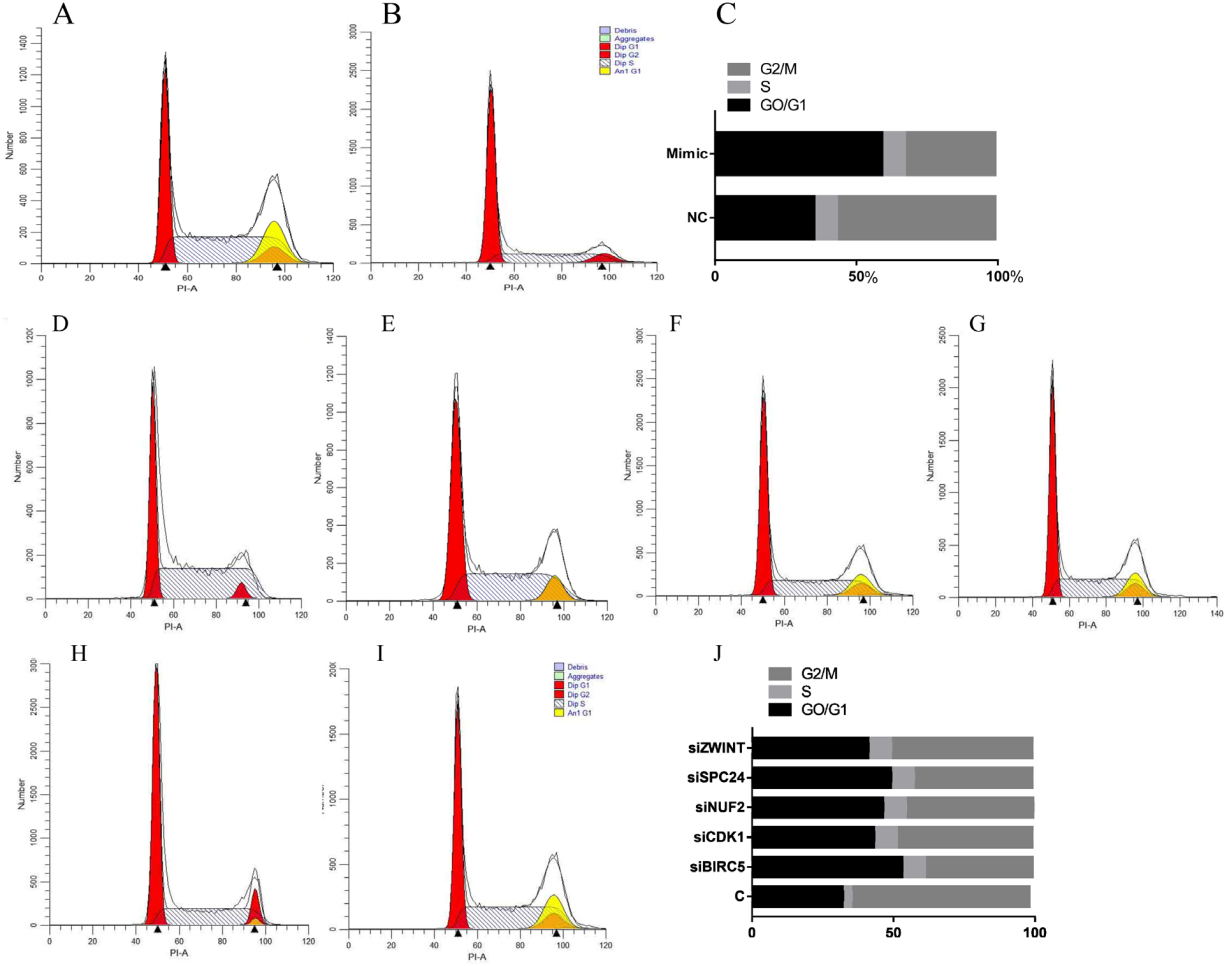
Cell cycle analysis detected by flow cytometry **(A, D)** Control group, **(B)** miRNA mimic group, **(C, J)** analysis of cell cycle data, **(E)** HepG2 treated with siBIRC5, **(F)** HepG2 treated with siCDK1, **(G)** HepG2 treated with siNUF2, **(H)** HepG2 treated with SPC24, **(I)** HepG2 treated with ZWINT. Data are presented as the mean ± SD. Compared with controls: ^*^P < 0.05.

Figure 9 showed that after transfection with hsa-miR-3613-3p mimics, the cells in G2/M phase decreased 57% compared with the negative control group, whereas cells in the G0/G1 phase increased 170% compared with the negative control group (P<0.05). Cells in G2/M decreased 67.9%, 76.2%, 71.4%, 66.6%, and 79.1% when transfected with siBIRC5, siCDK1, siNUF2, siSPC24 and siZWINT compared with the control group (P<0.05).

## DISCUSSION

In this study, we chose two profiles in the GEO database (GSE95698 and GSE41804), and screened 246 differentially expressed genes between liver cancer tissue and healthy control samples with GEO2R. Among them, 228 genes presented a similar trend in expression in both datasets. In these 228 DEGs, GO and KEGG pathway enrichment analysis were used. Integration of the PPI network and module analysis were used to screen 13 core genes, consisting of 12 up-regulated genes and 1 down-regulated gene. We predicted mRNA targets via miRWalk2.0 and found that hsa-miR-3613-3p was critical in reducing the expression level of 5 core genes. After cells were transfected, we determined that changed in hsa-miR-3613-3p were the most obvious. Therefore, we speculated that hsa-miR-3613-3p may be a potential target miRNA. Moreover, we transfected with si (BIRC5, CDK1, NUF2, ZWINT and SPC24), which can all be targeted by miR-3613-3p. Transfected HepG2 cells were tested for cell proliferation and migration. In conclusion, we hypothesized that hsa-miR-3613-3p, BIRC5, CDK1, NUF2, ZWINT, and SPC24 could affect the proliferation of HepG2 cells.

The liver regulates metabolic homeostasis by producing energy and molecules used by other cells in nearby or distant tissues. Liver cancer is ranked in the top ten of human cancers worldwide, and among the top five of cancers in terms of high mortality [5–7]. More than 70% of primary liver cancers [2, 8, 9] are presented as HCC [2, 9]. HCC is one of the most common causes of cancer-related death worldwide. Therefore, developing novel drugs to target HCC has become a major challenge and the focus of numerous studies. Cells go through a series of intracellular biochemical events to achieve proper growth, division of proliferation regulation, and to ensure that the cell cycle is sufficient. Abnormality of cell cycle regulation is the main mechanism of abnormal proliferation of tumor cells. Cell cycle misregulation plays a central role in promoting hepatocarcinogenesis through sustaining proliferative signaling.

In previous studies, BIRC5 has been reported in several malignant tumors. BIRC5 siRNA significantly inhibited cell proliferation in triple-negative breast cancer cells [11]. BIRC5 induced cancer cell apoptosis and cell cycle arrest, thereby efficiently inhibiting the proliferative activity in HCC [23]. BIRC5 protein expression in tumor tissue versus normal tissue was 1.3 times more often elevated in the squamous cell lung cancer group [24]. BIRC5 has been identified in tumorigenesis and progress of colorectal cancer (CRC). In addition, BIRC5 mRNA levels were significantly increased in serum of CRC patients, which indicates it potential to become a promising non-invasive biomarker for diagnosis of CRC [25]. The protein level of Ki67 and PCNA decreased after knocking down BIRC5, indicating that BIRC5 regulated the cell cycle of HepG2 cells. BIRC5 plays a crucial role in cell proliferation, adjusting the cell survival time, inhibiting the role of apoptosis, inhibiting the expression of the downstream product of BIRC5, and promoting cancer cell apoptosis in bladder tumor [26]. Anti-BIRC5 IgG could serve as a biomarker for the early diagnosis of cervical cancer [27].

Progress through the cell cycle is driven by members of the Cyclin-dependent kinase (CDK) family. As an important factor in CDK family, CDK1 is sufficient to drive the mammalian cell cycle. CDK1 was also found to regulate mitochondrial function, which improved cell cycle progression [28]. During the G2 to M transition, CDK1 activity levels impair DNA repair processes and play a major role in the yield of chromatid breaks induced after G2-irradiation [29]. Medicine induces G2/M phase arrest in human colorectal cancer colon 205 cells through inhibition of CDK1 activity [30]. Without synthesis of CDK1 prior to G2/M transition, the cell cannot enter mitosis, which leads to cell cycle arrest at the G2 phase. Interestingly, no significant differences were found in the protein levels of Ki67 and PCNA after the knockdown of CDK1. Similar findings were observed in the PCNA protein when cells were transfected with siZWINT. Ki67 and PCNA play a key role in the late G1 period, which explained that no changes were observed in Ki67 and PCNA levels.

NUF2 is part of a molecular linker between the kinetochore attachment site and tubulin subunits within the lattice of the attached plus ends [18]. Previous studies have reported that NUF2 is associated with several human cancers. One study reported that knockdown of NUF2 suppressed pancreatic cancer proliferation *in vitro* and *in vivo* [18]. Except for the testis, dysregulation of NUF2 has been reported in the development of several human cancers, including lung cancer, colorectal cancer, prostate cancer, as compared to normal tissue [31, 32]. Depletion of NUF2 resulted in the inhibition of cell proliferation in non-small-cell carcinoma and ovarian cancer [33]. In this study, protein levels of PCNA were significantly down-regulated after NUF2 interference. No significant changes in Ki67 protein were observed, however CCK-8 assay and flow cytometry did identify a difference.

PCNA is a nuclear protein associated with the cell cycle, and PCNA protein levels can be used as a marker to study cell proliferation. There is a clear correlation between up-regulation of PCNA expression and increased cell proliferation [34, 35]. In this study, the protein level of PCNA indicated that PCNA expression was visibly reduced after transfection with siBIRC5, siNUF2, or siSPC24, suggesting that the elevated PCNA expression participated in BIRC5, NUF2, and SPC24-induced cell proliferation.

Therefore, we suggested that BIRC5, NUF2, and SPC24 may be promising biomarkers in human liver cancer that provide information not only for predicting disease occurrence but also for suggesting personalized treatment options.

## MATERIALS AND METHODS

### Microarray analysis

Two human gene expression profiles (GSE95698 and GSE41804) were obtained. The array data of GSE95698 included 20 pairs of samples. GSE41804 contained data from 3 pairs of cancer and non-cancerous tissues analyzed by Affymetrix Human Genome U133 Plus 2.0 Array.

### Data processing

The GEO database archives a large number of high throughput functional genomic studies that contain data that are processed and normalized using various methods. GEO2R (http://www.ncbi.nlm.nih.gov/geo/geo2r/) was used to screen differentially expressed genes between CRC and healthy samples. GEO2R performs comparisons on original submitter-supplied processed data tables using the GEO query and limma R packages from the Bioconductor project. Adjusted P values (adj. P) were applied to correct for the occurrence of false positive results using the Benjamini and Hochberg false discovery rate method by default. The adj. P < 0.05 and |logFC| > 1 were set as the cut-off criteria.

### Functional and pathway enrichment analysis

The DAVID (https://david.ncifcrf.gov/home.jsp) provides researchers with a comprehensive set of functional annotation tools to understand the biological meaning behind a large list of genes. GO and KEGG pathway enrichment analysis were performed for identified DEGs using the DAVID database. P<0.05 was set as the cut-off criteria.

### Integration of protein-protein interaction network and module analysis

The functional interactions between proteins can provide context in molecular mechanism of cellular processing. In the current study, the PPI network of DEGs was constructed using the Search Tool for the Retrieval of Interacting Genes (STRING,http://string-db.org) database and was visualized using Cytoscape (http://www.cytoscape.org/index.html). A confidence score > 0.4 was set as the cut-off criteria. Subsequently, the MCODE was performed to screen modules of the PPI network with a degree cutoff = 2, node score cutoff = 0.2, K-Core = 2, and Depth from Seed = 100.

### Prediction of mRNA targets

MiRWalk2.0 (http://zmf.umm.uni-heidelberg.de/apps/zmf/mirwalk2/miRretsys-self.html) is freely accessible and comprehensive archived. MiRWalk2.0 provides the largest available collection of predicted and experimentally-verified mRNA-target interactions with various novel and unique features to assist the mRNA research community. MiRWalk2.0 is an integrated resource produced by established mRNA target prediction programs. The genes predicted by miRWalk, RNA22, miRanda, and Targetscan programs were identified as the targets of mRNAs (Table 5).

### Analysis of core protein expression in human liver cancer

Core protein expression in liver cancer tissue and normal tissue was evaluated using the human protein atlas (www.proteinatlas.org) [36].

### Cell culture and transfection

Human liver cancer cell HepG2 were cultured in RPMI 1640 medium (Life Technologies Inc., Cergy, Pontoise, France) supplemented with 10% fetal bovine serum (FBS; Gibco, Grand Island, NY, USA) and 1% penicillin/streptomycin (P/S; Sigma, St. Louis, MO, USA). In addition, HepG2 cells were supplemented with human insulin (0.1 U/mL) and incubated at 37°C in a humidified atmosphere of 5% CO_2_. HepG2 cells were plated in 6-well plates (5 × 10^5^ cell/well) for 24 h at 37°C, and transfected with hsa-miR-3613-3p mimic, hsa-miR-3613-3p, inhibitor, negative control, or small inhibitory RNA (siRNA) against core genes and NC-siRNA (RIBOBIO, Guangzhou, China) using Lipo2000 (Applied Biosystems, Life technologies, USA). Cells were harvested from 12 hours to 48 hours after transfection with miRNA or siRNA.

### Dual luciferase activity assay

To evaluate the binding specificity, we established a wild-type and mutant seed region of core genes (NUF2, BIRC5, CDK1, ZWINT, and SPC24) 3′UTR, which contained the putative target site for the mature hsa-miR-3613-3p, and cloned this into the pGL3-control vector. HepG2 cells were co-transfected with hsa-miR-3613-3p mimic, inhibitor, or negative control, pGL3 - core genes 3′UTR-WT vector, pGL3-core genes 3′UTR-MT vector, and phRL-SV40 control vector (Promega, USA) using Lipo2000 (Applied Biosystems, Life Technologies, USA) in 24-well plates. After 24 hours of transfection, the relative luciferase activity was determined by a Dual-Luciferase Reporter Assay System (Promega, USA) and normalized with renilla luciferase activity using SpectraMax i3 (Molecular Devices, USA).

### CCK-8 assay

Cell proliferation was determined by the Cell Counting Kit-8 (CCK-8). After 24 hours of transfection with miRNA or siRNA, HepG2 cells (1.0 × 10^3^) were plated in 96-well plates and cultured for 1, 2, 3, or 4 days, after with the supernatant was removed and the absorbance was measured at a wavelength of 450 nm.

### Real-time RT-PCR

Total RNA was extracted from tissues and cells using Trizol reagent (Applied Biosystems, Invitrogen, USA). The total RNA was reverse transcribed (Takara Bio Inc., Shiga, Japan) according to the manufacturer's guidelines. Primer analysis software (Oligo 7.24, Molecular BiologyInsights, Inc., USA) was used to design specific oligonucleotide primers (Table 4). Quantitative real-time RT-PCR was performed using an ABI 7900 (Life Technologies). Reactions were performed in 20 μL of reaction mixture, containing 10 μL PCR master mix (SYBR Premix Ex Taq II; Takara Bio Inc.), 0.4 μL primer pairs, and 2 μL cDNA. After normalizing to the expression of GAPDH, the relative expression levels of hsa-miR-3613-3p and core genes were calculated by the 2^−ΔΔCt^ method.

**Table 4:** List of mRNA primer sequences

Primer name	Accession	Forward Primer (5′-3′)	Reverse Primer (5′-3′)
BIRC5	CR541740.1	CGACGTTGCCCCCTGCCTGG	GACGACCCCATAGAGGAACAT
CCNB1	NM_031966.3	AACATGGCAGGCGCAAAGCGC	ACCTATGCTGGTGCCAGTGC
CDK1	NM_001320918.1	ATGGAAGATTATACCAAAATAGA	ATAGTCAGTCTTCAGGATGT
CENPE	NM_001286734.1	GAATCACTTGGAGAAACTGCCCA	CTACAATGGTACTATATTTG
DLGAP5	NM_001146015.1	AATAGACATAAGGAATACGAACG	AAAGCAACTTCAAAAATTGAAA
KIF18A	NM_031217.3	CCCAAACAAGAAGAAGTCAGTTT	CCTATGGTGCCACTGGTGCTGG
KIF20A	NM_005733.2	TGTTTGAGTCCACAGCTGCAGAT	CACCCAAGGACTCTTTTGCC
NUF2	NM_145697.2	CAAGAATGATCTTTATCCAAATCCA	TTTAGTTACTCATCTGGACT
PTTG1	CR541685.1	GCTGGGGTCTGGACCTTCA	AAGATGACTGAGAAGACTGTTA
SPC24	NM_001317031.1	TGCTGGGCGCCAACCGCGCGGA	AGCTGGAAGCTGGGCTTCAG
TOP2A	NM_001067.3	TTTTGTTCCTGGTTTGTACAA	GGTGGTCGAAATGGCTATGGAG
ZWINT	NM_001005413.1	AGCCAAGATCCTGGTTGAGTTT	AGGCCCTGACTCAGATGGAGGA

**Table 5:** Seed regions of miRNA and complementary 3′UTR region of mRNAs

mRNA name	Predicted consequential pairing of miRNA	Position site of mRNA 3′ UTR
BIRC5	UUUUUUG	237-243
CDK1	UUUUUGA	476-482
NUF2	ACAAAAA	1023-1031
SPC24	UUUUUUGA	97-104
ZWINT	UUUUUUG	698-704

### Western blot analysis

Protein extracts were subjected to SDS-polyacrylamide gel electrophoresis under reducing conditions using 15% gels. Proteins were then transferred to nitrocellulose membranes using tank transfer for 1.5 h at 200 mA in Tris-glycine buffer containing 20% methanol. The membranes were blocked with 5% skim milk for 18-24 h and incubated overnight at 4°C with primary antibodies directed against Ki67 and PCNA (1:1000, Santa Cruz Biotechnology, USA), followed by an anti-rabbit horseradish peroxidase (HRP)-conjugated secondary antibody IgG (1: 2000, Santa Cruz Biotechnology, USA). To verify equal sample loading, membranes were incubated with a monoclonal anti-GAPDH antibody (1: 1000, Santa Cruz Biotechnology, USA), followed by an HRP-conjugated goat anti-mouse IgG (1: 1000, Santa Cruz Biotechnology, USA). Proteins were visualized by and enhanced chemiluminescence system (Cheml Scope5300, Clinx Science Instruments, Shanghai, China).

### Cell cycle detection

Another challenge is the quantitative analysis of cell cycle by flow cytometry. Briefly, after treatment with miRNA and siRNA for 24h, the cells were washed twice with cold PBS and re-suspended in binding buffer at a concentration of 3 × 10^6^ cell/mL according to the instructions provided with the cell cycle detection Kit (BD Biosciences). The cells were incubated for 30 min at room temperature in the dark and analyzed by flow cytometry within 1 h after completion of the staining.

### Statistical analysis

All statistical parameters were calculated using GraphPad Prism 7.0 software. Values are expressed as the mean ± standard deviation (SD). Comparisons of two groups were performed using a Student's t-tests, more than 2 independent groups were compared using one-way analysis of variance (ANOVA). P < 0.05 was considered statistically significant. Ranking of genes by the degree of differential expression was performed by heat map analysis using the Heml 1.0 (http://hemi.biocuckoo.org/down.php).
